# Two-Dimensional Template Matching (2DTM) enables accurate identification and probing of macromolecular structure within *in situ* cryo-EM datasets.

**DOI:** 10.1063/4.0000958

**Published:** 2025-10-27

**Authors:** Matthew Giammar, Joshua Dickerson

**Affiliations:** Prof Bronwyn Lucas sUC Berkeley, Berkeley, CA, USA

## Abstract

Cryogenic electron microscopy (cryo-EM) and tomography (cryo-ET) are powerful tools to visualize biological structure and heterogeneity at the molecular level. Analyzing *in situ* data, as opposed to purified in vitro samples, is complicated by the crowded and low-contrast nature of cellular environments. Two-dimensional template matching (2DTM) is an emerging *in situ* cryo-EM data analysis technique sensitive enough to locate and orient molecules within cells based on a reference template. Importantly, 2DTM maintains contextual information allowing inference into biologically relevant structural heterogeneity, native interactions, and cellular localization. Here, I will cover 2DTM as a cryo-EM data analysis method focusing on improvements and extensions the Lucas Lab has recently made to 2DTM. For example, we have developed a new method called MOSAICS (Molecular *in **s***itu **A**tomic **C**oordinate Scanning) as a quantitative method for comparing reference structures with experimental cryo-EM data. We identify significant differences of <10 kDa with MOSAICS between populations of less than 150 molecules in the cell—two orders of magnitude fewer than required for classical 3D reconstruction— thereby enabling structural characterization of low abundance macromolecular states in situ. These advancements have been enabled by a new software package the lab has developed: Leopard-EM (**L**ocation and Orientati**o**n of Particles found using Two-**D**imensional Template **M**atching), an extensible and open-source Python package for GPU-accelerated. Creating flexible and performant software tools like Leopard-EM and MOSAICS, which build upon 2DTM, will help advance the fields of cryo-EM and in situ structural biology.

